# An all-optical tunable polymer WGM laser pumped by a laser diode†

**DOI:** 10.1039/d2na00025c

**Published:** 2022-03-30

**Authors:** Ben Niu, Xiaoyu Shi, Kun Ge, Jun Ruan, Zhiyang Xu, Shuai Zhang, Dan Guo, Tianrui Zhai

**Affiliations:** College of Physics and Optoelectronics, Faculty of Science, Beijing University of Technology Beijing 100124 China trzhai@bjut.edu.cn

## Abstract

An all-optical tunable whispering gallery mode (WGM) laser pumped by a laser diode is proposed. The laser is fabricated by filling a silica capillary with a light-emitting conjugated polymer solution. Based on the thermo-optic effect of the hydroxyl groups in the polymer and capillary, the effective refractive index of the WGM cavity changes by the auxiliary irradiation of the laser, and the wavelength of the WGM mode shifts correspondingly. The emission wavelength was continuously tuned over 13 nm with the irradiation power intensity changing from 0 to 22.41 W cm^−2^, showing a corresponding tuning rate of 0.58 nm W^−1^ cm^−2^. The wavelength tuning process has a fast response time that is within 2.8 s. It shows strong stability, with the output intensity showing no obvious attenuation after 100 minutes of operation. The proposed laser exhibits good repeatability, stability and high tuning efficiency, and could be applied as a light source for on-chip devices.

## Abstract 2

An all-optical tunable whispering gallery mode laser pumped by a laser diode is proposed. The emission wavelength was continuously tuned over 13 nm. The wavelength tuning process has fast response time that is within 2.8 s.

## Graphical abstract

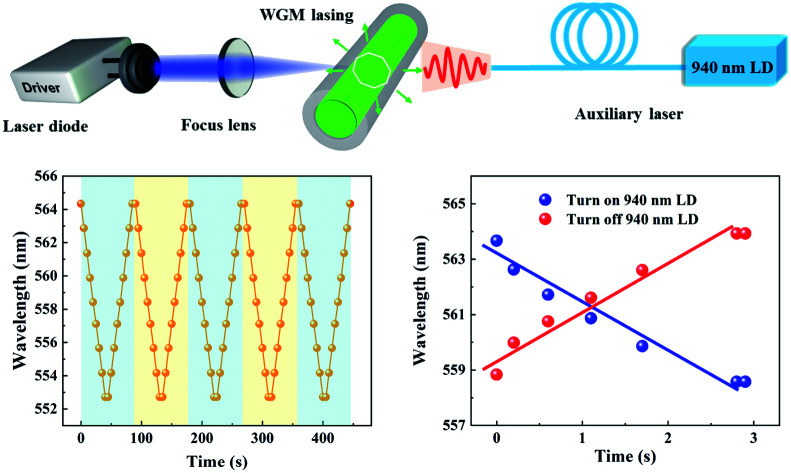

## 1. Introduction

Polymer lasers, owing to their advantage of a large wavelength tuning range,^1^ have become an important category of tunable laser sources over the whole visible spectrum, and have been applied in sensing^2–5^ and full color laser displays.^6–10^ The polymer materials possess the advantages of easy processing and low cost, which make them ideal gain media for lasers. In recent years, various geometries of polymer lasers have received significant attention, such as Fabry–Perot structures,^11^ distributed feedback structures,^12–14^ photonic crystals,^15,16^ and whispering-gallery-mode (WGM) cavities.^17–20^ However, a significant limitation of the current polymer lasers is the optical pump source. More recently, polymer lasers have been optically pumped by laser diodes (LDs) or by light emitting diodes,^21–26^ optimizing the compact and undoubtedly low-cost polymer laser systems, which greatly promotes their applicability.

The WGM microcavity resonators with a small mode volume and high quality (*Q*) factor are ideal cavities for polymer lasers that are pumped by LD or by light emitting diodes. Currently, various methods for achieving the wavelength tuning of WGM lasers have been reported,^27–32^ such as strain, injecting liquid, and temperature. Chen *et al.* have developed an optically pumped mechanically tunable WGM laser from polymer microfiber, demonstrating that bending is a powerful way for tuning the wavelength of WGM laser.^29^ Li *et al.* proposed an all-optical tunable whispering gallery mode (WGM) laser from a liquid-filled hollow glass microsphere.^32^ A secondary laser at 793 nm was used to irradiate the liquid-filled hollow glass microsphere to excite the NaNdF_4_ in the liquid core, resulting in a maximum tuning range of 4.95 nm and a sensitivity of 2.95 nm W^−1^ mm^−2^. Zhang *et al.* have reported a tunable WGM laser based on the polymer thermo-optic effect.^20^ Benefiting from the strong thermo-optic effect of polymers, the emission wavelength can be continuously tuned to about 19.5 nm as the temperature changes from 24 °C to 42.2 °C. Niu *et al.* have proposed a tunable polymer WGM laser pumped by a laser diode.^23^ By changing the concentration of the gain material, the laser wavelength is tuned over 15 nm. These results not only shed light on the good tunability of WGM lasing devices but also open up an avenue for the design of new tunable WGM lasers pumped by LD or by light emitting diodes.

In this study, we present an all-optical tunable WGM laser pumped by a laser diode. The device is prepared by filling a polymer solution into a capillary tube. A typical WGM laser emission is observed experimentally after excitation by a commercial laser diode at 450 nm. Based on the thermo-optic effect of the hydroxyl in the polymer and capillary tube, wavelength-tunable WGM lasing with high speed tuning response and wide tuning range is achieved by controlling the power intensity of the 940 nm irradiation laser diode. The reversibility and repeatability are studied, the polymer WGM laser shows a good spectral reversibility after five cycles of auxiliary power intensity. Furthermore, the device exhibits strong stability after 100 minutes of operation. Our work will provide a potential tunable light source for all-optical on-chip devices.

## 2. Fabrication of the polymer WGM laser

**Fig. 1:**
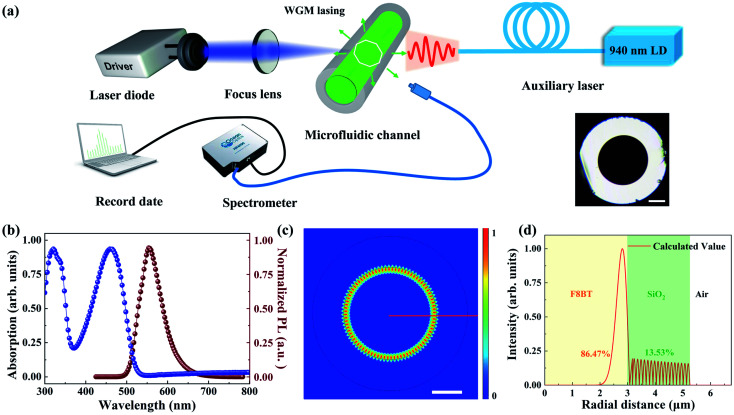
(a) The schematic diagram of the all-optical tunable diode laser pumped polymer WGM laser. The inset is the cross section of the capillary tube and the scale bar is 100 μm. (b) Absorption (blue line) and photoluminescence (brown line) spectra of F8BT, with its absorption peak around 450 nm. (c) Top view of the electric field distribution (TE_0_) of the polymer microcavity, the scale bar is 2 μm. The white and black circles denoted the boundaries of the capillary tube. (d) Simulated intensity profile (TE_0_) of the capillary tube cross section along the red line in (c).

The schematic of the laser diode-pumped all-optical tunable polymer WGM laser is shown in Fig. 1(a). A commercial silicon capillary tube (Zhong Cheng Quartz Glass Co., Ltd Beijing, China) with an inner diameter of 300 μm and wall thickness of 115 μm was selected as the microresonator. The optical micrograph of the cross section of the capillary tube is shown in the inset of Fig. 1(a) and the scale bar is 100 μm. A typical light-emitting polymer poly[(9,9-dioctylfluorenyl-2,7-diyl)-*alt-co*-(1,4-benzo-(2,1′,3)thiadiazole)] (F8BT, American Dye Source) was employed as the active material and is dissolved in xylene with concentrations of 12.5 mg mL^−1^. The absorption and photoluminescence (PL) characteristics of F8BT are shown in Fig. 1(b). Non-polarized white light from a tungsten halogen lamp (HL-2000) was used to characterize the absorption spectrum. There is a little overlap between the absorption and the PL spectrum, indicating that the self-absorption of the PL emission is very weak. When the capillary is dipped into the organic polymer solution, the solution will be imbibed into the capillary tube *via* the capillary effect. After that, the tube is dipped in a solution composed of polyvinyl alcohol (PVA, Sigma-Aldrich) dissolved in deionized water at a concentration of 40 mg mL^−1^, which acts as a sealant.

The lasing mechanism in the dye filled capillary is studied by numerically simulating the field distribution in the microcavity using the COMSOL software. We established a cylindrical shell structure with three layers. The refractive indices of air, tube wall, and dye solution were set as 1, 1.45, and 1.55, respectively. Based on the scaling law, the wall thickness and inner diameter of the capillary were set as 2.3 μm and 6 μm, respectively. All parameters are consistent with the experimental parameters. Fig. 1(c) presents the electric field distributions of a fundamental transverse-electric mode (TE_0_) in the microcavity. Fig. 1(d) exhibits the intensity profile of the microcavity cross section along the red line in Fig. 1(c). Almost all the energy (about 86.47%) is confined in the cavity, which is formed by silica and liquid polymer. Also, only a small portion spreads into the silicon layer of the microcavity, which provides the possibility for the realization of high-*Q* WGM lasing.

## 3. Spectra characterization of the WGM laser

A commercial laser diode (NDB7Y75, Nichia Corporation) with the maximum continuous output power of 5 W and central emission wavelength of 450 nm was used as the pump source. The threshold current of the laser diode is 0.32 A and slope efficiency is 1.8 W A^−1^. The astigmatic and highly divergent output beam is collimated and focused to an elliptical spot size with a diameter of 200 μm and 50 μm, respectively, along the vertical and horizontal direction. A pulsed power supply (Picolas GmbH) was used to generate laser pulses with a pulsewidth of 50 ns at a repetition rate of 200 Hz. When such short pulsewidth is used, the laser diode can be driven at high peak current levels of up to 30 A, resulting in an optical pulse power intensity of 0.53 MW cm^−2^ in the microcavity. A fiber-coupled laser diode (DF01H4948T, Skyralaser, China) operating at 940 nm, which is matched with the absorption peak of the hydroxyl (OH),^33^ is used to trigger photo-thermal effects. The output fiber is fixed at 11 mm away from the capillary microcavity. A spectrometer (HR 4000, Ocean Optics) with a spectral resolution of 0.01 nm was used to collect the emission of the WGM lasers.

**Fig. 2:**
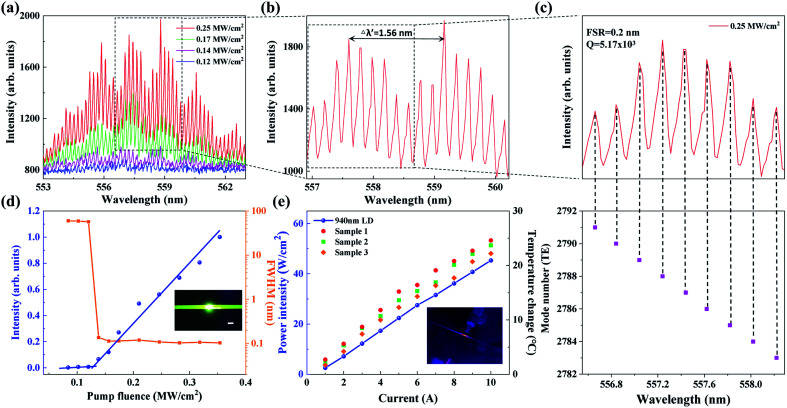
(a) The lasing spectra of the diode laser-pumped polymer WGM microcavity collected with increased pump power density. (b) Enlarged view of the WGM lasing mode clusters. (c) The enlarged view of the lasing spectra from the polymer WGM laser. The corresponding mode number is from 2783 to 2791. (d) The output peak intensities (the blue circle with linear fitting) and linewidths (orange squares connected with a line) at the wavelength of 558.8 nm as a function of the pump energy. Inset: photograph of the excited WGM laser, with a scale bar of 50 μm. (e) The output power intensity and temperature rise of 3 samples with increased pump current of the auxiliary fiber-coupled 940 nm diode laser. Inset: infrared photograph of the polymer microcavity irradiated by a 940 laser diode.

The evolution of the PL spectrum of the laser diode-pumped WGM laser is demonstrated when the pumping power density ranges from 0.12 MW cm^−2^ to 0.25 MW cm^−2^, as shown in Fig. 2(a). Only a broad spontaneous emission spectrum peak is observed when the pumping peak power density is lower than 0.12 MW cm^−2^. Also, the FWHM is over 50 nm. When the pump power density is raised above 0.14 MW cm^−2^, the weak broad spectrum transformed into sharp peaks centered at about 558 nm. Also, a significant increase in the emission peak intensity is observed. These features indicate that the resonant feedback is built up in the polymer-filled capillary microcavity. An enlarged view of the peak spacing at a pump power density of 0.25 MW cm^−2^ is shown in Fig. 2(b). The value of the free spectral range (FSR) is 0.2 nm. The equation for the WGM laser can be illustrated as:1
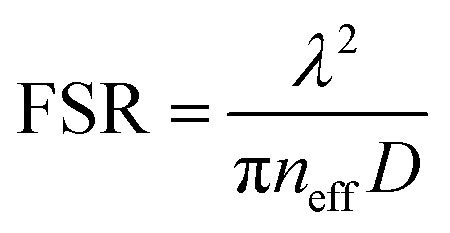
where *λ* is the wavelength, and *n*_eff_ is the effective refractive index, and *D* is the inner diameter of capillary.

It should be noted that the intensity of the emission spectra decreases periodically in Fig. 2(b), forming some mode clusters. The spacing between adjacent mode clusters Δ*λ* is 1.56 nm caused by the two-ring coupling effect.^20^ The capillary tube consists of two mismatched cylinder microrings, forming a coupled asymmetric microcavity, which causes inhibition between the two nearby ring microcavities. Fig. 2(c) presents a typical WGM lasing spectrum with lasing peaks that match well with mode numbers from 2783 to 2791 of the first order TE modes. The mode numbers are calculated according to the WGM theory:

2 *mλ*_m_ = π*n*_eff_*D*

where *λ*_m_ is the peak wavelength and *n*_eff_ is the effective refractive index of the polymer solution, and *D* is the inner diameter of the capillary. In our study, the value of *Q* can be estimated as 5.17 × 10^3^ by the equation *Q* = *λ*/Δ*λ*, where *λ* is 558 nm and Δ*λ* is 0.108 nm. A plot of the intensity and FWHM of the emission spectra as a nonlinear function of the pump power density are depicted in Fig. 2(d), and exhibit a clear lasing threshold at 0.128 MW cm^−2^. The related image showing lasing from the polymer WGM laser is presented in the inset of Fig. 2(d). The laser output power density and polymer microcavity temperature rise as a function of the drive current, as shown in Fig. 2(e). The inset is a photograph of the 940 nm laser-irradiated polymer microcavity taken with a thermal imager (875-1, Testo, Germany).

When the laser diode at 940 nm is irradiated into the polymer-filled capillary microcavity, the photo-thermal effect changes the effective refractive index and diameter of the microcavity, thereby altering the resonant wavelength. The temperature-induced change in the resonance wavelength (Δ*λ*) can be expressed as follows:^34,35^3
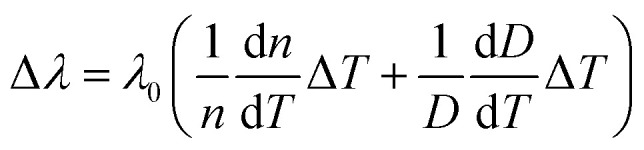
where d*n*/d*T* and d*D*/d*T* are the thermo-optic and thermal expansion coefficients, respectively. *λ*_0_ is the resonant emission wavelength at room temperature, and Δ*T* is the value of the temperature change of the polymer-filled microcavity. The thermo-optic coefficient for the polymer dissolved in xylene is −3.0 × 10^−3^. The small thermal expansion coefficient (about 10^−7^) of the silica capillary imposes constraint on the thermally induced expansion of the polymer-filled capillary microcavity. The geometric parameter changes of the microcavity can be ignored. Therefore, the resonant wavelength is mainly affected by the temperature change of the polymer-doped xylene for achieving the all-optical tunable laser diode-pumped WGM lasing.

**Fig. 3:**
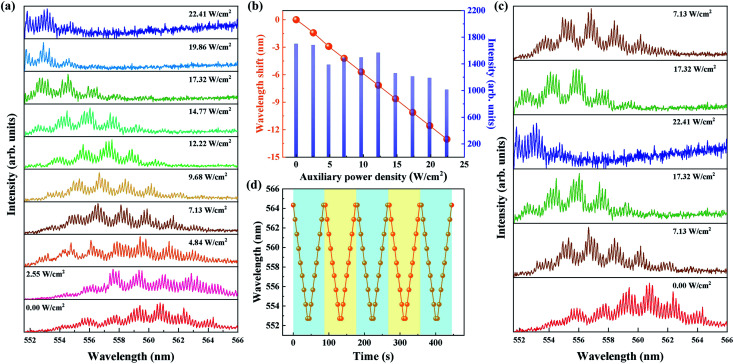
(a) Lasing spectra of the diode laser-pumped polymer capillary WGM laser (with pump power density of 0.35 MW cm^−2^ and repetition frequency of 200 Hz) *versus* power density of a fiber coupled 940 nm laser diode. (b) Wavelength shift and intensity change in the polymer capillary WGM lasing as a function of the 940 nm laser power density, respectively. (c) Lasing spectrum as a function of cycled laser power density. (d) Reversibility of the lasing wavelength when 940 nm laser power density increases and decreases periodically from 0 to 19.86 W cm^−2^.

The tuning characteristics of the laser diode-pumped polymer WGM laser were investigated. When the 940 nm laser is manipulated to irradiate into the polymer capillary microcavity, a series of typical WGM spectra are obtained. As shown in Fig. 3(a), with the increase in the output power density of the 940 nm laser diode from 0 to 22.4 W cm^−2^. As the temperature of the polymer liquid rises, the lasing peaks exhibit a continuous blue shift due to the negative thermo-optic coefficient. Fig. 3(b) shows that the total wavelength shift is up to 13 nm without a decrease in the emission intensity.

The reverse process of tuning characteristics of the polymer WGM laser was also investigated. There is a redshift with the decrease in the output power density. The lasing spectra for a complete cycle of the 940 nm laser output power density was investigated, as shown in Fig. 3(c). The function between the lasing wavelength and the time was investigated, while the power density of the 940 nm laser diode increases and decreases periodically. Nine power density values used in Fig. 3(a) (except for 22.41 W cm^−2^) are selected and cycled in 100 seconds. Their stable lasing wavelength are each recorded simultaneously. The results of five cycles are shown in Fig. 3(d). The results demonstrate that the lasing wavelength usually returns to the original value when the power density is reset, which implies that the laser diode-pumped polymer capillary WGM laser has good spectral reversibility.

**Fig. 4:**
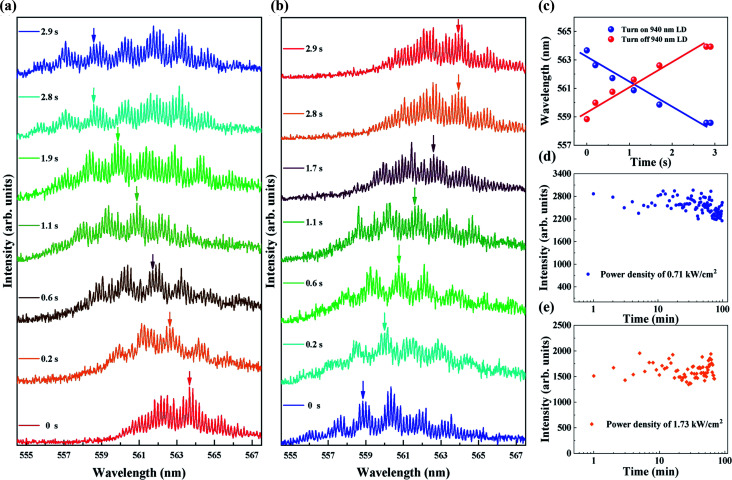
(a) The dynamic evolution of the wavelength blue-shifts when the 940 nm laser diode is turned on. (b) The dynamic evolution of wavelength red-shifts when the 940 nm laser diode is turned off. (c) The linear function of wavelength and temporal variation when turning on and turning off the 940 nm laser diode. The evolution of the lasing spectral intensity with a pumping power density of 0.35 MW cm^−2^, (d) 100 minutes with the power density of the 940 nm laser diode at 7.13 W cm^−2^. (e) 70 minutes with the power density of 17.32 W cm^−2^.

The temporal tuning characteristics of the polymer capillary WGM laser were investigated with a pumping power density of 0.35 MW cm^−2^. A series of spectra were recorded by the spectrometer at 50 microsecond intervals. As shown in Fig. 4(a), the blue shift of the wavelength from the polymer microcavity is complete in 2.8 s when the 940 nm laser diode is turned on, which provides a power density of 9.68 W cm^−2^. Correspondingly, the wavelength red shift is complete in 2.8 s when the laser diode is turned off, as shown in Fig. 4(b). The relationship of the wavelength shift and irradiation time was investigated for a specific mode. The results shown in Fig. 4(c) indicate that the wavelength shifts linearly with irradiation time.

The stability of the all-optical tunable laser diode-pumped polymer WGM laser was also investigated in our study. When the laser diode pumping power density is 0.35 MW cm^−2^, two power densities of 7.13 and 17.32 W cm^−2^ are used to irradiate the polymer capillary. The intensity of the emission was recorded every minute and is shown in Fig. 4(d) and (e), respectively. After 100 minutes and 70 minutes of operation, respectively, the emission intensity has no obvious attenuation, indicating that the polymer capillary WGM laser has high stability and can be used as a potential on-chip light source.

## 4. Conclusions

In summary, we have realized an all-optical tunable WGM laser in a polymer-filled capillary tube based on laser diode pumping. Based on the photo-thermal effect of the hydroxyl in the polymer and capillary, the lasing wavelength of the polymer WGM laser can be tuned up to 13 nm by controlling the power density of a 940 nm irradiating laser diode. Moreover, the output lasing density of the device is not significantly weakened after 130 minutes under an irradiation power density of 17.32 W cm^−2^. The experimental results indicate that the all-optical tunable polymer WGM laser has good stability and reversibility, which makes the laser diode pumped laser a promising candidate for on-chip light source.

## Author contributions

Conceptualization, Tianrui Zhai and Dan Guo; investigation, Xiaoyu Shi and Ben Niu; methodology, Kun Ge and Shuai Zhang; project administration, Zhiyang Xu; resources, Jun Ruan; writing, Ben Niu. All authors have read and agreed to the published version of the manuscript.

## Conflicts of interest

There are no conflicts to declare.

## Supplementary Material

## References

[cit1] Samuel I. D. W., Turnbull G. A. (2004). Polymer lasers: recent advances. Mater. Today.

[cit2] Wang Y., Zeng S., Humbert G., Ho H. (2020). Microfluidic whispering gallery mode optical sensors for biological applications. Laser Photonics Rev..

[cit3] Vollmer F., Arnold S. (2008). Whispering-gallery-mode biosensing: label-free detection down to single molecules. Nat. Methods.

[cit4] Yang Y., Saurabh S., Ward J., Chormaic S. (2016). High-Q, ultrathin-walled microbubble resonator for aerostatic pressure sensing. Opt. Express.

[cit5] Lu Q., Liao J., Liu S., Wu X., Liu L., Xu L. (2016). Precise measurement of micro bubble resonator thickness by internal aerostatic pressure sensing. Opt. Express.

[cit6] Zhao J. Y., Yan Y. L., Gao Z. H., Du Y. X., Dong H. Y., Yao J. N., Zhao Y. S. (2019). Full-color laser displays based on organic printed microlaser arrays. Nat. Commun..

[cit7] Ge K., Guo D., Ma X. J., Xu Z. Y., Hayat A., Li S. T., Zhai T. R. (2021). Large-area biocompatible random laser for wearable applications. Nanomaterials.

[cit8] Lv Y. C., Li Y. J., Li J., Yan Y. L., Yao J. N., Zhao Y. S. (2017). All-color subwavelength output of organic flexible microlasers. J. Am. Chem. Soc..

[cit9] Ge K., Shi X. Y., Xu Z. Y., Cui L. B., Guo D., Li S. T., Zhai T. R. (2021). Full-color WGM lasing in nested microcavities. Nanoscale.

[cit10] Yu H. Q., Liao D. W., Johnston M. B., Li B. J. (2011). All-Optical Full-Color Displays Using Polymer Nanofibers. ACS Nano.

[cit11] Dai H. L., Yin C., Xiao Z. Y., Cao Z. Q., Chen X. F. (2019). White beam lasing from a hybrid microcavity with slab-capillary mode coupling. Phys. Rev. Appl..

[cit12] Kim J. H., Inoue M., Zhao L., Komino T., Seo S., Ribierre J. C., Adachi C. (2015). Tunable and flexible solvent-free liquid organic distributed feedback lasers. Appl. Phys. Lett..

[cit13] Doring S., Kollosche M., Rabe T., Stumpe J., Kofod G. (2011). Electrically Tunable Polymer DFB Laser. Adv. Mater..

[cit14] Foucher C., Guilhabert B., Herrnsdorf J., Laurand N., Dawson M. D. (2014). Diode-pumped, mechanically-flexible polymer DFB laser encapsulated by glass membranes. Opt. Express.

[cit15] Fu Y. L., Zhai T. R. (2020). Distributed feedback organic lasing in photonic crystals. Front. Optoelectron..

[cit16] Burratti L., De Matteis F., Casalboni M., Francini R., Pizzoferrato R., Prosposito P. (2018). Polystyrene photonic crystals as optical sensors for volatile organic compounds. Mater. Chem. Phys..

[cit17] Gu F. X., Xie F. M., Lin X., Linghu S. Y., Fang W., Zeng H. P., Tong L. M., Zhuang S. L. (2017). Single whispering-gallery mode lasing in polymer bottle microresonators *via* spatial pump engineering. Light: Sci. Appl..

[cit18] Wang H. X., Liao M. M., Xiao H. F., Zhang Z. F., Yang J. B., Yang J. H., Tian Y. H. (2021). All-Optical Tunable Whispering Gallery Modes in a Polymer Bottle Micro-Resonator. IEEE Photonics Technol. Lett..

[cit19] Lu Q. J., Wu X., Liu L. Y., Xu L. (2015). Mode-selective lasing in high-Q polymer micro bottle resonators. Opt. Express.

[cit20] Zhang S., Zhai T. R., Cui L. B., Shi X. Y., Ge K., Liang N. N., Hayat A. (2021). Tunable WGM Laser Based on the Polymer Thermo-Optic Effect. Polymers.

[cit21] Stefanska D., Suski M., Zygmunt A., Stachera J., Furmann B. (2019). Tunable single-mode CW energy-transfer dye laser directly optically pumped by a diode laser. Opt. Laser Technol..

[cit22] Tsiminis G., Wang Y., Kanibolotsky A. L., Inigo A. R., Skabara P. J., Samuel I. D. W., Turnbull G. A. (2013). Nanoimprinted polymer semiconductor laser pumped by a light-emitting diode. Adv. Mater..

[cit23] Niu B., Ge K., Xu Z. Y., Shi X. Y., Guo D., Zhai T. R. (2021). Laser Diode Pumped Polymer Lasers with Tunable Emission Based on Microfluidic Channels. Polymers.

[cit24] Zhao Z., Mhibik O., Nafa M., Chenais S., Forget S. (2015). High brightness diode-pumped polymer solid-state laser. Appl. Phys. Lett..

[cit25] Burdukova O., Gorbunkov M., Petukhov V., Semenov M. (2017). Diode pumped tunable dye laser. Appl. Phys. B.

[cit26] Wang Y., Morawska P. O., Kanibolotsky A. L., Skabara P. J., Turnbull G. A., Samuel I. D. W. (2013). LED pumped polymer laser sensor for explosives. Laser Photonics Rev..

[cit27] Toropov N., Cabello G., Serrano M. P., Gutha R. R., Rafti M., Vollmer F. (2021). Review of biosensing with whispering-gallery mode lasers. Light: Sci. Appl..

[cit28] Zhao L. Y., Wang Y., Yuan Y. G., Liu Y. J., Liu S. Q., Sun W. M., Yang J., Li H. Y. (2017). Whispering gallery mode laser based on cholesteric liquid crystal microdroplets as temperature sensor. Opt. Commun..

[cit29] Chen R., Ta V. D., Sun H. D. (2014). Bending-Induced Bidirectional Tuning of Whispering Gallery Mode Lasing from Flexible Polymer Fibers. ACS Photonics.

[cit30] Liu S. Q., Shi B. J., Sun W. M., Li H. Y., Yang J. (2018). Whispering gallery mode resonance transfer in hollow microcavities. Appl. Phys. Express.

[cit31] Yuan Z. Y., Tan X. T., Gong X. R., Gong C. Y., Cheng X., Feng S. L., Fan X. D., Chen Y. C. (2021). Bioresponsive Microlasers with Tunable Lasing Wavelength. Nanoscale.

[cit32] Li H. Y., Hao X. L., Li Y. Z., Xu L., Shi B. J., Liu L. (2020). Nanoheater-tuned whispering gallery mode lasing in liquid-filled hollow microcavities. Opt. Lett..

[cit33] Keck D. B., Maurer R. D., Schultz P. C. (1973). On the ultimate lower limit of attenuation in glass optical waveguides. Appl. Phys. Lett..

[cit34] Genscha T., Viappiani C. (2003). Time-resolved photothermal methods: accessing time-resolved thermodynamics of photoinduced processes in chemistry and biology. Photochem. Photobiol. Sci..

[cit35] Wan L., Chandrahalim H., Chen C., Chen Q., Mei T., Oki Y., Nishimura N., Guo L. J., Fan X. (2017). On-chip, high-sensitivity temperature sensors based on dye-doped solid-state polymer microring lasers. Appl. Phys. Lett..

